# The European Reference Network for Rare Neurological Diseases

**DOI:** 10.3389/fneur.2020.616569

**Published:** 2021-01-14

**Authors:** Carola Reinhard, Anne-Catherine Bachoud-Lévi, Tobias Bäumer, Enrico Bertini, Alicia Brunelle, Annemieke I. Buizer, Antonio Federico, Thomas Gasser, Samuel Groeschel, Sanja Hermanns, Thomas Klockgether, Ingeborg Krägeloh-Mann, G. Bernhard Landwehrmeyer, Isabelle Leber, Alfons Macaya, Caterina Mariotti, Wassilios G. Meissner, Maria Judit Molnar, Jorik Nonnekes, Juan Dario Ortigoza Escobar, Belen Pérez Dueñas, Lori Renna Linton, Ludger Schöls, Rebecca Schuele, Marina A. J. Tijssen, Rik Vandenberghe, Anna Volkmer, Nicole I. Wolf, Holm Graessner

**Affiliations:** ^1^Institute for Medical Genetics and Applied Genomics, University of Tübingen, Tübingen, Germany; ^2^Centre for Rare Diseases, University Hospital Tübingen, Tübingen, Germany; ^3^Assistance Publique-Hôpitaux de Paris, National Reference Center for Huntington's Disease, Neurology Department, Henri Mondor-Albert Chenevier Hospital, Créteil, France; ^4^Département d'Etudes Cognitives, École normale supérieure, PSL University, Paris, France; ^5^Inserm U955, Institut Mondor de Recherche Biomédicale, Equipe E01 NeuroPsychologie Interventionnelle, Créteil, France; ^6^Institute of Systems Motor Science, University of Lübeck, Lübeck, Germany; ^7^Centre for Rare Diseases, University of Lübeck, Lübeck, Germany; ^8^Unit of Neuromuscular and Neurodegenerative Disorders and Genetics and Rare Diseases Research Division, Bambino Gesù Children's Research Hospital, Instituto de Ricovero e Cura a Carattere Scientifico, Rome, Italy; ^9^Department of Rehabilitation Medicine, Amsterdam Movement Sciences and Emma Children's Hospital, Amsterdam University Medical Centers, Vrije Universiteit Amsterdam, Amsterdam, Netherlands; ^10^Department of Medicine, Neurology, and Neurosciences, Medical School, University of Siena, Siena, Italy; ^11^Department of Neurodegenerative Diseases, Hertie Institute for Clinical Brain Research, University of Tübingen, Tübingen, Germany; ^12^German Center for Neurodegenerative Diseases, Tübingen, Germany; ^13^Department of Paediatric Neurology and Developmental Medicine, University Children's Hospital, Tübingen, Germany; ^14^Department of Neurology, University Hospital Bonn, Bonn, Germany; ^15^University of Ulm, Department of Neurology, Ulm, Germany; ^16^Sorbonne Universités, Paris Brain Institute – Institut du Cerveau – ICM, Inserm U1127, CNRS UMR 7225, AP-HP - Hôpital Pitié-Salpêtrière, Paris, France; ^17^Reference Centre for Rare or Early Dementias, IM2A, Département de Neurologie, AP-HP - Hôpital Pitié-Salpêtrière, Paris, France; ^18^Pediatric Neurology Department, Vall d'Hebron Research Institute and Neuroscience Institute, Autonomous University Barcelona, Barcelona, Spain; ^19^Unit of Medical Genetics and Neurogenetics, Fondazione Instituto de Ricovero e Cura a Carattere Scientifico Istituto Neurologico Carlo Besta, Milan, Italy; ^20^CRMR AMS, Service de Neurologie des Maladies Neurodégénératives, CHU Bordeaux, France and Univ. Bordeaux, CNRS, IMN, UMR 5293, Bordeaux, France; ^21^Department of Medicine, University of Otago, New Zealand Brain Research Institute, Christchurch, New Zealand; ^22^Institute of Genomic Medicine and Rare Disorders, Semmelweis University, Budapest, Hungary; ^23^Department of Rehabilitation, Centre of Expertise for Parkinson and Movement Disorders, Donders Institute for Brain, Cognition and Behaviour, Radboud University Medical Centre, Nijmegen, Netherlands; ^24^Movement Disorders Unit, Institut de Recerca Sant Joan de Déu, and Centro de Investigación Biomédica en Red de Enfermedades Raras Instituto de Salud Carlos III (CIBERER-ISCIII), Barcelona, Spain; ^25^Department of Pediatric Neurology, Hospital Vall d'Hebrón, Pediatric Neurology Research Group at Vall d'Hebrón Research Institute, Universitat Autonoma de Barcelona, Barcelona, Spain; ^26^EuroHSP, Plateforme Maladies Rares, Paris, France; ^27^Department of Neurology, Hertie Institute for Clinical Brain Research, University of Tübingen, Tübingen, Germany; ^28^Department of Neurodegenerative Diseases, Center of Neurology, Hertie Institute for Clinical Brain Research, University of Tübingen, Tübingen, Germany; ^29^Expertise Center Movement Disorders Groningen, University Medical Center Groningen, University of Groningen, Groningen, Netherlands; ^30^Neurology Service, University Hospitals Leuven, Leuven, Belgium; ^31^Laboratory for Cognitive Neurology, Department of Neurosciences, Katholieke Universiteit Leuven, Leuven, Belgium; ^32^Division of Psychology and Language Sciences, University College London, London, United Kingdom; ^33^Department of Therapy Services, University College London Hospitals National Health System Foundation Trust National Hospital for Neurology and Neurosurgery, London, United Kingdom; ^34^Department of Child Neurology, Amsterdam Leukodystrophy Centre, Emma Children's Hospital, Amsterdam University Medical Centres, Vrije Universiteit, and Amsterdam Neuroscience, Amsterdam, Netherlands

**Keywords:** rare neurological diseases, standards of care, training and education, virtual healthcare, European reference network

## Abstract

While rare diseases (RDs) are by definition of low prevalence, the total number of patients suffering from an RD is high, and the majority of them have neurologic manifestations, involving central, peripheral nerve, and muscle. In 2017, 24 European Reference Networks (ERNs), each focusing on a specific group of rare or low-prevalence complex diseases, were formed to improve the care for patients with an RD. One major aim is to have “the knowledge travel instead of the patient,” which has been put into practice by the implementation of the Clinical Patient Management System (CPMS) that enables clinicians to perform pan-European virtual consultations. The European Reference Network for Rare Neurological Diseases (ERN-RND) provides an infrastructure for knowledge sharing and care coordination for patients affected by a rare neurological disease (RND) involving the most common central nervous system pathological conditions. It covers the following disease groups: (i) Cerebellar Ataxias and Hereditary Spastic Paraplegias; (ii) Huntington's disease and Other Choreas; (iii) Frontotemporal dementia; (iv) Dystonia, (non-epileptic) paroxysmal disorders, and Neurodegeneration with Brain Iron Accumulation; (v) Leukoencephalopathies; and (vi) Atypical Parkinsonian Syndromes. At the moment, it unites 32 expert centers and 10 affiliated partners in 21 European countries, as well as patient representatives, but will soon cover nearly all countries of the European Union as a result of the ongoing expansion process. Disease expert groups developed and consented on diagnostic flowcharts and disease scales to assess the different aspects of RNDs. ERN-RND has started to discuss diagnostically unclear patients in the CPMS, is one of four ERNs that serve as foundation of Solve-RD, and has established an RND training and education program. The network will facilitate trial readiness through the establishment of an ERN-RND registry with a minimal data of all patients seen at the ERN-RND centers, thus providing a unique overview of existing genotype-based cohorts. The overall aim of the ERNs is to improve access for patients with RDs to quality diagnosis, care, and treatment. Based on this objective, ERNs are monitored by the European Commission on a regular basis to provide transparency and reassurance to the RD community and the general public.

## Introduction

### Rare Diseases in Europe—The Current Situation and Challenges

In Europe, a disease is considered as “rare” if it affects <1 person in 2000 (1). Although rare diseases (RDs) have–per definition–a low prevalence, the total number of patients with an RD is high, concerning about 3.5–5.9% of the population that equates to 263–446 million persons affected globally at any point in time (2). The majority of RD have neurological manifestations, involving central, peripheral nerve and muscle (3). Most RDs are associated with high unmet needs due to the lack of available and effective diagnosis and treatment measures as well as the relative lack of research to develop such measures, at least partly due to the low number of medical experts available for each condition and limited financial resources. A current analysis of Orphanet has shown that of the 5,304 diseases defined by point prevalence, 84.5% of those analyzed have a point prevalence of <1/1,000,000 (2) and can thus be characterized as ultra-rare diseases. This means that no single European Union (EU) member state can provide access to the best possible healthcare to its citizens in all areas of highly specialized healthcare for RD patients on its own.

### European Reference Networks for Rare Diseases

As response to this challenge, European Reference Networks (ERNs) were launched in 2017 by the European Union Board of Member States as a pan-European initiative to facilitate access to highly specialized healthcare for patients with rare or low-prevalence complex diseases. It also aims to reinforce the cooperation of Healthcare Providers in the field of RDs at the European level.

ERNs are legally based on the European Directive 2011/24/EU on patients' rights in cross-border healthcare.

This worldwide-unique initiative resulted in 24 ERNs being formed, involving more than 900 specialized healthcare units from over 300 hospitals in 26 Member States (4). There are three ERNs with a neurological focus: ERN EpiCARE^1^ on rare epilepsies, Euro-NMD^2^ on rare neuromuscular diseases, and European Reference Network for Rare Neurological Diseases (ERN-RND) that will be described in more detail below.

Each ERN had to fulfill a number of criteria for implementation, evaluation, and knowledge sharing, while the respective national authorities endorsed the individual healthcare providers to become an ERN member. For a detailed description of the conceptual framework, see Heon-Klin (5).

The central political aim of the ERNs is that medical expertise “travels,” and that only in a few cases (e.g., for highly specialized interventions and for diagnostic and therapeutic measures that are not available in the country the respective person lives in) the patient has to travel. This marks a significant step toward improving healthcare quality, harmonizing medical (diagnosis) procedures, reducing access inequalities, and increasing overall medical experience and knowledge in the whole of Europe.

In addition, the ERNs open the possibility to get sizable cohorts of patients in the perspective of therapeutic trials and the development of research collaborations.

### Travel of Knowledge Put Into Practice—The Clinical Patient Management System

To improve the diagnosis and treatment of RDs in practical terms, the European Commission has set up the Clinical Patient Management System (CPMS^3^), a web-based clinical software application, enabling secure remote multi-national and multidisciplinary case discussions.

The CPMS can be used by healthcare professionals of all ERNs, who can upload patient data in a structured manner following an informed consent procedure. Clinicians from outside the ERNs can request CPMS-based advice from ERNs on specific patients through referring them to the nearest national ERN healthcare provider. So far, over 40 CPMS case discussions have been performed by ERN-RND members.

### The European Reference Network for Rare Neurological Diseases

The ERN-RND is a network of the European RND expertise centers. At present, it has 32 full members and 10 affiliated partners from 21 countries (a list of the actual ERN-RND full members and affiliated partners, their countries, and their respective areas of expertise are provided as Supplementary Material); however, through a currently ongoing expansion process, the ERN-RND will be including the vast majority of EU countries by mid-2021. Thus, ERN-RND will be the first truly pan-European rare neurological disease (RND) network that brings together all respective European expertise centers. Governance and activities of ERN-RND are patient centered that is reflected by the active involvement of European Patient Advocacy Groups (ePAGs) representatives.

The formation of ERN-RND is timely since RNDs present a topic of continuously growing importance in neurology. Rapid advances in clinical knowledge in recent years have been facilitated by the emergence of genetic and other diagnostic technologies, helping us to develop a deeper understanding of RDs and their causes. The defined genetic etiology of the majority of RNDs has, moreover, been facilitating the development of targeted molecular therapies for RND, such as viral vector-based gene therapy and antisense oligonucleotides.

The huge heterogeneity of RND and healthcare systems in Europe, as well as the still limited clinical expert workforce base for RND at a time of rapid clinical innovation, means that there is a very real risk that significant parts of estimated more than 500,000 RND patients across Europe might not benefit from improved diagnosis, care, and treatment opportunities.

Consequently, the objectives of the ERN-RND are as follows:

- To significantly increase the overall percentage of RND patients with a confirmed (molecular) diagnosis
- To improve and harmonize care including neurorehabilitation and transition of RND patients across the EU
- To develop, share, and implement care pathways and guidelines for all RND groups represented in ERN-RND
- To create, develop, and enhance the capacity to design, implement, and supervise RND training, education, and capacity building activities at the level of member states and of the network
- To develop comprehensive and data-based European RND cohorts to be able to deploy digital solutions, including artificial intelligence-based tools, for diagnosis and patient-centered integrated care, in order to better understand these conditions and thus improve their management and help developing and testing treatments
- To define the minimum quality and interoperability criteria for RND registries allowing the exchange of data between existing registries and the ERN-RND registry.

Building on existing mature RND European disease networks, such as the European Huntington's Disease Network^4^, the Ataxia Study Group^5^, and DystoniaNet^6^, which have already strong clinical collaborations, ERN-RND focuses on the following disease groups at the moment:

1) Cerebellar Ataxias and Hereditary Spastic Paraplegias (Coordinators: Enrico Bertini, Alfons Macaya, Caterina Mariotti, Rebecca Schuele)
2) Huntington's disease and Other Choreas (Coordinators: Anne-Catherine Bachoud-Lévi, Bernhard Landwehrmeyer, Juan Dario Ortigoza Escobar)
3) Frontotemporal dementia (Coordinators: Isabelle Leber, Markus Otto, Rik Vandenberghe)
4) Dystonia, (non-epileptic) paroxysmal disorders, and Neurodegeneration with Brain Iron Accumulation (Coordinators: Tobias Bäumer, Belen Pérez Dueñas, Giovanna Zorzi)
5) Leukoencephalopathies (Coordinators: Odile Boespflug-Tanguy, Ingeborg Krägeloh-Mann, Samuel Groeschel, Nicole I. Wolf).
6) Atypical Parkinsonian Syndromes (Coordinators: Thomas Gasser, Wassilios Meissner).

**Figure 1:**
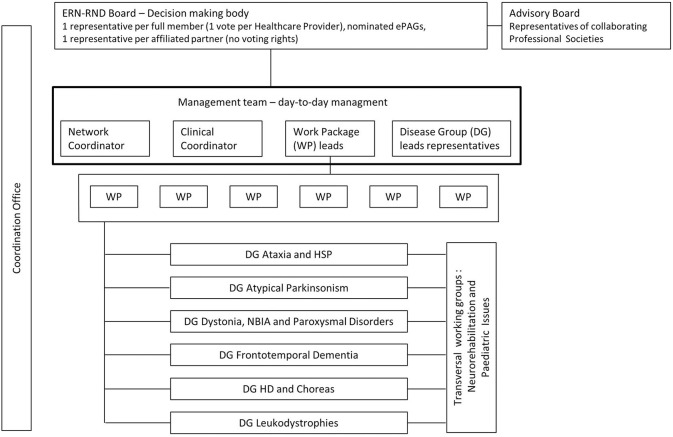
Network and governance structure of ERN-RND.

ERN-RND is coordinated at the University Hospital Tübingen, Germany, with Holm Graessner being the coordinator and Ludger Schöls the clinical lead. An overview about the network and governance structure of ERN-RND is given in Figure 1.

Activities of ERN-RND are coordinated based on the covered disease groups as well as the following cross-cutting lines of work that address the objectives:

1) RND diagnostic pathways (Coordinator: Alexandra Durr)
2) Expert RND Care Coordination (Coordinator: Marina de Koning-Tijssen)
3) Training, education, and capacity building (Coordinator: Maria Judit Molnar)
4) Information sharing and disease resources (Coordinator: Holm Graessner)
5) Guidelines, pathways, and best practice (Coordinator: Antonio Federico)
6) Registries and Research (Coordinator: Thomas Klockgether)
7) Pediatric issues (Coordinators: Juan Dario Ortigoza Escobar, Caroline Sevin, Nicole I. Wolf)
8) Neurorehabilitation (Coordinators: Annemieke I. Buizer, Antonio Federico, Maria Judit Molnar, Jorik Nonnekes, Lori Renna Linton).

## RND Diagnostic Pathways

To improve the diagnosis of RND patients, ERN-RND has been implementing four activities. Firstly, the visibility of the ERN-RND expertise centers has been improved through collaboration with Orphanet, by providing contact information on a newly created website^7^ and by rolling out a comprehensive multi-channel information campaign. Secondly, disease knowledge documents have been created and are being provided to the different stakeholder groups. In particular, disease expert groups developed and consented on RND diagnostic flowcharts, as well as on disease scales to assess the different aspects of RND. ERN-RND recommends the use of these flowcharts and disease scales and actively disseminates them to clinicians, national and European professional societies, and patient organizations^8^. Thirdly, ERN-RND has started to discuss patients without a definitive diagnosis in the CPMS. Fourthly, ERN-RND has been collaborating with the EU rare disease diagnostic research flagship project Solve-RD (Solving the unsolved Rare Diseases^9^); ERN-RND is one of four ERNs that serve as foundation of Solve-RD.

## Expert RND Care Coordination

RND care needs and situations differ between disease groups as well as between countries. In order to capture and assess care needs ERN-RND can address, a care need survey has been performed for all disease groups covered by ERN-RND across all EU countries (6). The survey is based on a respective publication for dystonia (7). Main measures to improve the management of RND patients that have been identified include the (i) development of multidisciplinary teams, (ii) implementation of educational activities to enhance recognition of RND among healthcare professionals and in the general population, (iii) improvement of the accessibility to standard and advanced genetic testing and to clinical geneticists, and (iv) development of more dedicated tertiary centers meaning more expertise in the field.

In addition, treatment algorithms and the composition of the multidisciplinary care team for all covered RND will be developed and consented on. By this process and the activities regarding diagnostic flowchart and assessment scales, ERN-RND will help to develop a comprehensive knowledge body of RND care standards within ERN-RND.

## Training, Education, and Capacity Building

ERN-RND has implemented an RND training and education program^10^ based on the following pillars:

1) Educational webinar series in collaboration with the European Academy of Neurology (EAN) and the European Reference Network for Neuromuscular Disorders (Euro-NMD).
2) Hands-on training winter and summer schools for young neurologists past topics ranging from “Diagnostic of rare movement disorders” to “Hereditary white matter diseases—clinics, genetics, therapy”.
3) Short-term mobility fellowships for RND healthcare professionals.

In particular, the webinar series has attracted a high number of attendees both across Europe and globally and has been expanded from topics based on disease knowledge to neurorehabilitation.

## Information Sharing and Disease Resources

ERN-RND aims to become the information hub for all available information for the diseases covered by the network. Therefore, ERN-RND collects and edits available information as well as produces new information and knowledge. Disease knowledge, to be included in ERN-RND disease knowledge pages, needs to undergo an affirmation process that includes patients' representatives as well as expert clinicians from the network. ERN-RND reached out to 27 European national neurological societies with regard to setting up an RND webpage on their respective society website to increase the visibility of RNDs across the European community of neurologists. Although this has not yet been implemented, it is an important part of our awareness strategy. In addition to this, the ERN-RND monthly newsletter is sent to a varied audience including patients, patient advocates, clinicians, and researchers in Europe to inform them about the latest developments within the network and in the RND field in general. All disease information is compiled on the ERN-RND website^11^. Efforts are provided to multiply document access to patients from different languages.

## Guidelines, Pathways, and Best Practice

Clinical practice guidelines for RD are scarce and difficult to find (8). Therefore, ERN-RND will use the expertise of its partners to adopt and develop clinical practice guidelines for the diseases covered by the network. GRADE methodology^12^ will be used, following the recommendations by the EAN (9, 10). The work on European guidelines for diagnosis and treatment of Metachromatic Leukodystrophies has recently started.

Furthermore, ERN-RND endorses or affirms the value of existing guidelines, depending on whether an EAN equivalent methodology was used or not. Examples are: Management of the ataxias toward best clinical practice (11) and the International Guidelines for the Treatment of Huntington's Disease (12).

As a matter of fact, guideline development in RND often struggles with the lack of systematic reviews and strong evidence. ERN-RND actively contributes to the project of the EAN on guidance for developing and reporting guidelines in the field of RNDs, as well as the critical appraisal of all existing RND guidelines.

Furthermore, in the context of the Value of Treatment project^13^, ERN-RND has set up a collaboration with the European Brain Council to assess the benefits of expertise centers for rare neurological disorders in consideration of the quality of care being provided and cost-effectiveness.

## Registries and Research

With the foundation of ERN-RND, a large network of potential trial sites for RND has been formed. As the ERN-RND expertise centers are very likely those centers that are performing and will perform RND treatment trials in Europe, the network will facilitate trial readiness through three activities. Firstly, it is about to establish an ERN-RND registry that is going to comprise minimal data of all patients seen at the ERN-RND centers. This registry will thus provide a unique overview of existing genotype-based cohorts. The minimal data set being used is based on the “Set of common data elements for Rare Diseases Registration” as recommended by the European Platform on Rare Disease Registration^14^. Secondly, ERN-RND has organized and will be organizing multi-stakeholder workshops focusing on the different aspects of trial readiness. Thirdly, ERN-RND will strive to support trial readiness platforms, such as ARCA and SCA Global^15^, in order to help in addressing major knowledge gaps that preclude further progress toward the development of effective therapies in RNDs.

## Pediatric Issues

The working group on pediatric issues has been formed recently to specifically address the different needs of pediatric RND. The cross-cutting pediatric issues should be addressed (i) within ERN-RND, (ii) in collaboration with other ERNs, and (iii) linked with the European Pediatric Neurological Society (EPNS).

As a first step, a mapping exercise is being performed on the specific neuropediatric expertise of the ERN-RND centers as well as on existing pediatric scales that are used across the disease groups to identify potential gaps. Patient information leaflets with a focus on pediatric issues as well as information about clinical trials should be collected and made available on the ERN-RND website. In addition, collaboration with the EPNS, e.g., focusing on joint training activities, has been implemented.

## Neurorehabilitation

As causal treatments are only scarcely available for RND, neurorehabilitation is an important aspect in the management of these diseases. To address this need, a specific working group has been formed with the following goals: (i) organization of teaching courses on neurorehabilitation of the different RNDs. As a first step, online training webinars focusing on neurorehabilitation of RND have been organized. (ii) Organization of national/regional networks for RND neurorehabilitation. (iii) Guidelines production in collaboration with EAN panels and the European Federation for Neurorehabilitation.

As a first step, a mapping of the locally used neurorehabilitation protocols including the evidence on which they are based and the possibility of transfer to other centers is underway.

## Monitoring and Evaluation of ERN-RND

The overall aim of the ERNs is to improve access for patients with RDs to quality diagnosis, care, and treatment. Based on this objective, ERNs are monitored on a regular basis by the European Commission to guarantee transparency and provide reassurance of both the RD community and the general public. Additional reasons for monitoring include quality improvement, accountability, and identification of needs for strategy adjustments and promotion of patient empowerment.

## Discussion and Conclusions

ERN-RND provides an expertise-based infrastructure for sharing knowledge and coordinating care for patients affected by RNDs. The evolving network presents a new unique organization able to create, take up, and implement emerging diagnostic, care, and treatment innovations.

Built as a virtual network, ERN-RND provides–besides the flagship CPMS–also e-solutions for all other areas of cooperation of the different project bodies and stakeholders, including webinars, cloud-based document-repositories, and web conferences.

Future challenges include equity of quality of care being provided across the EU as well as the systematic integration of ERN into the national healthcare systems.

## Author Contributions

HG and CR are the coordinator and project manager, respectively, of the European Reference Network for Rare Neurological Diseases and wrote the publication. LS is the clinical lead of ERN-RND. SH and AB are members of the coordination office. RS, CM, EB, AM, RV, IL, SG, IK-M, NW, A-CB-L, BL, JO, TG, WM, BP, and TB are disease group coordinators. AF, MT, MM, TK, JO, AIB, JN, LR, and AV are working group coordinators. All authors are responsible for the work described in their respective project roles and critically reviewed the manuscript.

## Conflict of Interest

TG has received research support from the Deutsche Forschungsgemeinschaft (DFG), the Bundesministerium für Bildung und Forschung (BMBF), the European Union (EU), and the National Institutes of Health (NIH). He has received speakers honoraria from, Roche, Teva and UBC. SG received institutional research support from Shire plc, outside of the submitted work. He is advisor and co-investigator for trials in Metachromatic Leukodystrophy (Shire/Takeda, Orchard), but receives no personal payment related to this role. TK has received research support from the Deutsche Forschungsgemeinschaft (DFG), the Bundesministerium für Bildung und Forschung (BMBF), the Bundesministerium für Gesundheit (BMG), the Robert Bosch Foundation, the European Union (EU), and the National Institutes of Health (NIH). He has received consulting fees from Biohaven, Roche and UBC. He has received a speaker honorarium from Novartis and Bayer. IL served as a member of advisory boards for Prevail Therapeutic and received research grants from ANR, DGOS, Vaincre Alzheimer Association, ARSla Association, Fondation Plan Alzheimer outside of the present work. Unrelated to the present work, WM has received fees for editorial activities with Springer and Elsevier, has served as advisor for Lundbeck and Biohaven, and has received teaching honoraria from UCB. MT reports grants from the Netherlands Organization for Health Research and Development ZonMW Topsubsidie (91218013), the European Fund for Regional Development from the European Union (01492947) and the province of Friesland, Dystonia Medical Research Foundation, from Stichting Wetenschapsfonds Dystonie Vereniging, from Fonds Psychische Gezondheid, from Phelps Stichting, and an unrestricted grants from Actelion and AOP Orphan Pharmaceuticals AG. NW is advisor and co-investigator for trials in Metachromatic Leukodystrophy and other leukodystrophies (Shire/Takeda, Orchard, Ionis, PassageBio), but receives no personal payment related to this role. HG has received research support from the Deutsche Forschungsgemeinschaft (DFG), the Bundesministerium für Bildung und Forschung (BMBF), the Bundesministerium für Gesundheit (BMG) and the European Union (EU). He has received consulting fees from Roche. He has received a speaker honorarium from Takeda. GL receives/has received research support from Bundesministerium für Bildung und Forschung (BMBF), CHDI Foundation, Deutsche Forschungsgemeinschaft (DFG), the European HD Network (EHDN), and the European Union (EU - Horizon2020, JPND), GL serves/served at Scientific Advisory Boards of Hoffmann-LaRoche, Novartis, PTC Therapeutics, TEVA, Triplet Therapeutics & Takeda and provided scientific advice to Acadia Pharm, AOP Orphan, Boehringer-lngelhe1m, CHDI Foundation, lonis Pharma, Lundbeck, NeuraMetrix, Prilenia, and Wave. MM received research support from the National Research, Development and Innovation Office for the National Brain Research and the National Bionics Projects. She has received consulting fees from Richter Gedeon Plc, Novartis, Sanofi-Genzyme, PTC Therapeutics, Stealth Biotherapeutics, Amicus, Takeda, and Biogen. She has received a speaker honorarium from Johnson and Johnson and AOP Orphan. The remaining authors declare that the research was conducted in the absence of any commercial or financial relationships that could be construed as a potential conflict of interest.

## Footnotes

^1^https://epi-care.eu/

^2^https://ern-euro-nmd.eu/

^3^https://cpms.ern-net.eu/login/

^4^http://www.ehdn.org

^5^http://www.ataxia-study-group.net

^6^https://dystonia.net/

^7^www.ern-rnd.eu

^8^http://www.ern-rnd.eu/disease-knowledge-hub/.

^9^www.solve-rd.eu.

^10^http://www.ern-rnd.eu/education-training/

^11^www.ern-rnd.eu

^12^http://www.gradeworkinggroup.org/

^13^https://www.braincouncil.eu/activities/projects/the-value-of-treatment

^14^https://eu-rd-platform.jrc.ec.europa.eu/_en.

^15^http://ataxia-global-initiatives.net/.
